# Miliary Histoplasmosis in a Patient with Rheumatoid Arthritis

**DOI:** 10.1155/2018/2723489

**Published:** 2018-04-16

**Authors:** Jessica Lum, Maheen Z. Abidi, Bruce McCollister, Andrés F. Henao-Martínez

**Affiliations:** Division of Infectious Diseases, University of Colorado, Denver, CO, USA

## Abstract

Miliary histoplasmosis is a rare presentation that may mimic miliary tuberculosis. We report a case of miliary histoplasmosis in a 52-year-old male who was being treated with hydroxychloroquine, methotrexate, and sulfasalazine for his rheumatoid arthritis and presented to the emergency department with shortness of breath and fevers. Computed tomography (CT) chest revealed miliary pulmonary nodules. Urine *Histoplasma* antigen and serum *Histoplasma* antigen were negative; however, *Coccidioides immitis* complement immunofixation assay and *Coccidioides* IgM were positive. The patient was initiated on treatment for pulmonary coccidioidomycosis and immunosuppression was held. However, a few days later, *Histoplasma capsulatum* was isolated from cultures from bronchoscopy. This case highlights the difficulty in diagnosing histoplasmosis in immunocompromised patients and the importance of having a broad differential diagnosis for miliary pulmonary nodules. Tissue culture and histopathology remain the gold standard for the diagnosis of histoplasmosis. Further research needs to be conducted to determine the optimal duration of histoplasmosis treatment in immunocompromised patients.

## 1. Background

*Histoplasma capsulatum* is an environmental fungus found commonly in the Ohio and Mississippi River valleys in the United States, Central and South America, and Asia [1, 2]. Risk factors for infection include living or traveling to an endemic area, exposure to aerosolized soil that contains spores, and exposure to bats or birds and their droppings. Patients infected with histoplasmosis can have a wide range of clinical presentations. Many patients are asymptomatic or have a subclinical disease that resolves spontaneously. However, immunocompromised individuals, including HIV/AIDS patients, solid-organ transplant recipients, bone marrow transplant recipients, patients receiving tumor necrosis factor (TNF) inhibitors, and patients with cellular immune dysfunction are at increased risk for disseminated infection [3]. Miliary histoplasmosis is a rare clinical manifestation that may mimic miliary tuberculosis and diagnosis may be delayed if a broad differential is not considered in patients undergoing workup for miliary pulmonary nodules.

We describe a case of a male with rheumatoid arthritis (RA) on immunosuppressing medications who presented with fevers and shortness of breath and was found to have miliary histoplasmosis. In immunocompromised patients, diagnosis and management of histoplasmosis may be more challenging. Serum and urine *Histoplasma capsulatum* antigens may be negative even in the setting of active infection, and there is limited data on the duration of treatment for patients who remain immunocompromised.

## 2. Case Report

A 52-year-old man with a history of RA presented to the emergency department with a one-week history of fevers, chills, headache, nausea, vomiting, and diarrhea. He also reported having a nonproductive cough, fatigue, and one month of night sweats.

His therapy for RA comprised weekly methotrexate, daily hydroxychloroquine, and sulfasalazine. His symptoms were well-controlled on this regimen. He also gave a history of receipt of rituximab for a year prior to presentation.

Our patient is originally from Mexico but has lived in the United States for over forty years. Prior to moving to Colorado, he gave a history of residence in Ohio. He works as a truck driver and his route involves the Midwest states. He also gave a history of travel to California nearly a year ago. About one month before admission, he unloaded his truck in Kansas City during a rainstorm and shortly thereafter began having night sweats and a nonproductive cough. No known history of exposure to tuberculosis.

On admission, he was afebrile but was found to have an oxygen saturation of 85% on room air and required 2-3 liters/minute of supplemental oxygen. Initial laboratory studies revealed a normal white blood cell count. Liver enzymes were elevated above his baseline (AST 74 U/L, ALT 60 U/L, and alkaline phosphatase 170 U/L). An abdominal ultrasound showed mild hepatosplenomegaly, and a chest X-ray showed a diffuse reticulonodular density with hilar lymphadenopathy. He received one dose of intravenous (IV) ceftriaxone and azithromycin and was started on empiric treatment with IV ceftriaxone, doxycycline, and metronidazole for community-acquired pneumonia. Additional negative studies included a respiratory viral PCR, *Histoplasma* urine antigen, *Streptococcus pneumoniae* urine antigen, *Legionella* urine antigen, human immunodeficiency virus (HIV) 4th generation antibody/antigen assay, acute hepatitis panel, serum herpes simplex virus (HSV) PCR, serum cytomegalovirus (CMV PCR), *Mycobacterium tuberculosis* (MTB) quantiferon gold, and sputum *Pneumocystis jirovecii* pneumonia (PJP) direct fluorescent antibody (DFA). Rheumatology recommended continuing hydroxychloroquine and stopping methotrexate and sulfasalazine in the setting of possible infection.

**Figure 1:**
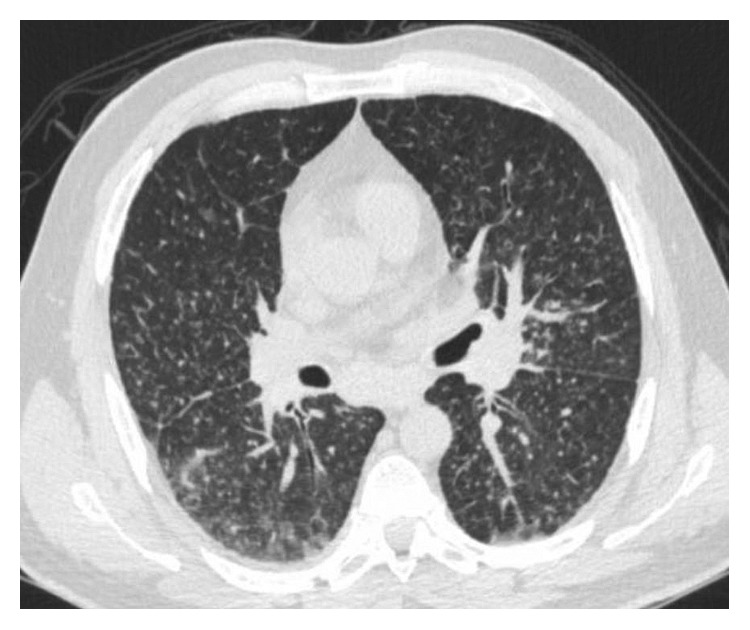
CT chest showing diffuse micronodules in all five of his lobes in a perilymphatic and centrilobular distribution, as well as a few scattered calcified granulomas.

A CT chest interstitial lung disease (ILD) showed diffuse micronodularity in all five pulmonary lobes in a perilymphatic and centrilobular distribution and few scattered calcified granulomas that suggested disseminated infection. The CT chest scan also showed mediastinal and hilar lymphadenopathy as well as splenomegaly (Figure 1). Diffuse micronodules had not been seen on previous CT chest scans six years prior and one month prior to presentation. His imaging findings suggested a miliary pattern. This combined with his clinical symptoms and environmental exposure history was concerning for an invasive fungal infection or tuberculosis (TB). Three sputum AFB cultures were collected and were smear negative. An MTB complex nucleic acid amplification test (NAAT) was performed on one of his AFB sputum samples and was not detectable.

He underwent bronchoscopy and findings were notable for endobronchial nodules and plaques in the left upper lobe. Multiple biopsies were done and sent for standard aerobic, AFB and fungal cultures. An *Aspergillus* galactomannan antigen and PJP DFA in bronchoalveolar lavage fluid (BAL) were negative. Since all cultures remained negative, his antibiotics were stopped after 3 days. Infectious Diseases was consulted and recommended starting empiric anidulafungin and posaconazole combination therapy given concern for a fungal infection. Empiric combination therapy with an azole + echinocandin was chosen to cover possible endemic fungi and invasive aspergillosis. Posaconazole was chosen over voriconazole for the addition of Mucorales coverage. Assays for serum *Histoplasma* antigen enzyme immunoassay (EIA), urine *Histoplasma* galactomannan antigen quantitative EIA, serum *Aspergillus* galactomannan antigen, and serum *Cryptococcus neoformans/gattii* antigen were negative. T-SPOT interferon-gamma release assay was negative. *Aspergillus fumigatus* IgG was elevated at 13.03 U/mL. Pathology from bronchoscopy biopsy showed necrotizing granulomatous inflammation suggestive of infectious etiology. Acid-fast bacilli (AFB) and Grocott-Gomori methenamine silver (GMS) stains were negative. Periodic acid– Schiff (PAS) stain and hematoxylin and eosin (HE) stain were not done. Five days after bronchoscopy, *Coccidioides* antibody by complement fixation (CF) assay returned positive at 1 : 8, *Coccidioides* IgM by enzyme-linked immunosorbent assay (ELISA) was positive, and *Coccidioides* IgG by ELISA was negative. *Coccidioides immitis* antibody by immunodiffusion (ID) demonstrated a partial identity reaction indicating the presence of two different types of antibodies. One antibody was identical to the positive control, and the other antibody was unrelated and could be a cross-reactive antibody due to infection with other systemic fungi. His *Coccidioides immitis* antibody was repeated three days later and was reported as detectable. This pattern of serological data suggested active coccidioidomycosis. He was started on liposomal amphotericin B. Posaconazole and anidulafungin were discontinued. After one week of liposomal amphotericin B treatment, he was transitioned to oral fluconazole 400 mg daily. His hypoxia resolved, and he was discharged home.

**Figure 2:**
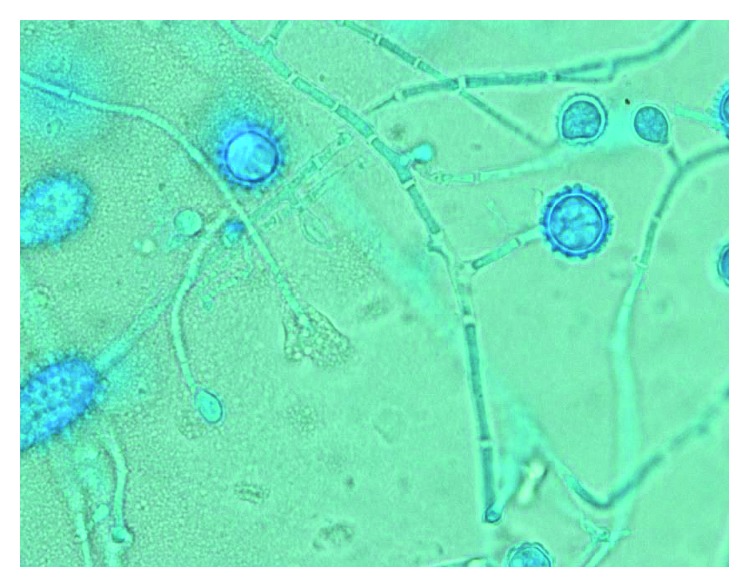
Tape preparation slide of *Histoplasma capsulatum* from left upper lobe tissue culture viewed at 100x magnification.

Nine days after discharge, our microbiology lab reported growth of mold from fungal lung tissue cultures. The isolate was preliminarily identified as *Histoplasma capsulatum* based on morphology (Figure 2) and was sent to a reference laboratory where it tested positive for *Histoplasma capsulatum* by DNA probe. A *Histoplasma capsulatum* DNA probe sent from his left upper lobe tissue culture was positive. On follow-up visit in the Infectious Diseases clinic, he was switched from fluconazole to itraconazole for the treatment of pulmonary histoplasmosis. A repeat chest CT showed improvement in the size and number of bilateral lung micronodules. One month after discharge, he reported resolution of shortness of breath. However, he described worsening of pain from RA in his shoulders, knees, wrists, and ankles. He was restarted on sulfasalazine and methotrexate after he had been on treatment for histoplasmosis for more than two months. Approximately eight months after presentation, his CT imaging continued to display multiple bilateral pulmonary nodules. *Histoplasma* antibodies by CF were checked and were negative (<1 : 8). He has been continued on his oral itraconazole treatment with plans to treat for at least one year.

## 3. Discussion

Miliary nodules are diffuse lung micronodules that resemble millet seeds on chest imaging [4]. They can occur in the setting of ineffective cellular defenses and hematogenous dissemination of diseases [5]. Both infectious and noninfectious causes should be in the differential diagnosis of miliary nodules. Noninfectious causes include sarcoidosis, hypersensitivity pneumonitis, pneumoconiosis, and metastatic cancer [5, 6]. Miliary tuberculosis is commonly associated with miliary pneumonia. Cases of miliary *Nocardia*, *Mycoplasma*, *Blastomyces* [7, 8], *Coccidioides* [9], *Cryptococcus* [10], and *Pneumocystis jirovecii* [11] have been reported.

Miliary histoplasmosis is a rare clinical presentation that can mimic tuberculosis. It should be considered when working up patients with miliary nodules of an infectious etiology. Only a few cases of miliary histoplasmosis have been described in the literature. A comparison of our case to other cases of miliary histoplasmosis is summarized in Table 1 [12–15].

Histoplasmosis infections can originate from acute de novo infections or following activation of clinically latent infections. Our patient previously lived in Ohio and had traveled to Kansas City shortly before his illness, and both locations are endemic areas where he may have acquired acute histoplasmosis. He was at higher risk for developing the infection as he was immunocompromised and on immunosuppressants for his RA. The other listed cases of miliary histoplasmosis that are listed in Table 1 involved young individuals who were not immunocompromised except for one patient with HIV [15].

Our patient was previously on etanercept, a TNF inhibitor, and rituximab for his rheumatoid arthritis but had not taken either medication for over a year. At the time of diagnosis, he was taking methotrexate, a disease-modifying antirheumatic drug (DMARD). Histoplasmosis is known to occur in patients with rheumatoid arthritis on DMARD therapies. In a 2011 review by Olson et al., 26 patients with rheumatoid arthritis who were seen at the Mayo Clinic were diagnosed with histoplasmosis, and the most common DMARD used was methotrexate [16].

He presented with symptoms of fever, cough, and night sweats similar to other patients with miliary histoplasmosis. In all the cases, imaging showed miliary shadows or diffuse micronodules consistent with a miliary pattern. Many patients were initially diagnosed with miliary tuberculosis, and some were started on empiric antituberculosis medications, including a patient who was clinically deteriorating [13]. Our patient was not started on antituberculosis medications while undergoing workup as he remained clinically stable. Unlike the other patients, he was initially diagnosed with miliary coccidioidomycosis based on positive coccidioidomycosis serologies and negative urine and serum *Histoplasma* antigens.

Histoplasmosis may be challenging to diagnose in immunocompromised patients. Diagnosis can be made by visualization of yeast on histopathology, isolation of the organism in culture, or detection of histoplasmosis antigens in body fluids [2]. Histopathology and culture are the gold standards for diagnosis. *Histoplasma capsulatum* can take at least 4 weeks to be detected in cultures, and DNA probes may be used to assist with diagnosis [2]. Our patient had tissue cultures from the left upper lobe which grew *Histoplasma capsulatum* after 3 weeks. His diagnosis of histoplasmosis was confirmed by a positive DNA probe sent from tissue culture. Pathology from his left upper lobe biopsy showed granulomatous inflammation which may be seen in *H. capsulatum* infection [2].

Antigen testing is noninvasive, and combining serum and urine antigen testing can increase sensitivity. The sensitivity of the antigen test is higher in more severe or disseminated infections [3, 17]. Serology may also help to make the diagnosis, but it may not be reliable in patients who cannot produce antibodies due to immune defects [3]. Our patient had negative *Histoplasma* antibodies by CF, and urine and serum *Histoplasma* antigens were measured and were negative despite tissue cultures growing *Histoplasma*. A 2011 multicenter study evaluated the sensitivity of serum and urine antigen assays in 218 patients with histoplasmosis. In immunocompromised patients with pulmonary histoplasmosis, the urine antigen test was positive in only 50% of patients [17]. In the report by Olson et al., only 13 of 24 patients diagnosed with histoplasmosis had positive urine antigens [16].

There is cross-reactivity of the *Histoplasma* antigen test with antigens from other endemic mycoses including *Blastomyces dermatitidis*, *Paracoccidioides brasiliensis*, *Coccidioides immitis*, and *Coccidioides posadasii* [18]. Cross-reactivity can occur with CF and ID assays in the setting of other fungal infections [18]. Kuberski et al. reviewed 19 cases of patients diagnosed with coccidiodomycosis who also underwent *Histoplasma* antigen testing. *Histoplasma* urinary antigen was positive in 11 (58%) of patients suggesting cross-reactivity between the two fungi [19]. Our patient had a positive *Coccidioides immitis* complement fixation and *Coccidioides* IgM. It was initially thought that the patient was infected with *Coccidioides*. However, the clinical course was more consistent with histoplasmosis, especially since his tissue culture grew *Histoplasma capsulatum*, and *Coccidioides* was not isolated in culture. Interestingly, his initial *Coccidioides immitis* antibody by ID demonstrated a partial identity reaction indicating two different types of antibodies were present. It is possible that one of the antibodies was due to cross-reactivity in the setting of histoplasmosis.

There are no randomized controlled studies that establish the optimal duration of treatment for pulmonary miliary histoplasmosis. Relapse of histoplasmosis after treatment is a valid concern, especially in immunocompromised patients. Studies assessing optimal treatment duration of histoplasmosis in immunocompromised patients have been limited to AIDS patients, solid-organ transplant recipients, and bone marrow transplant recipients. A 2014 multicenter retrospective cohort study by Myint et al. evaluated the Infectious Diseases Society of America (IDSA) guideline recommendations on when to discontinue antifungal therapy in patients with AIDS and histoplasmosis. The study concluded that antifungal therapy could be discontinued in patients who were adherent to therapy and completed at least 1 year of antifungal treatment, had a CD4 count >150 cells/mL, HIV RNA <400 copies/mL, *Histoplasma* urine antigen <2 ng/mL, and no CNS involvement [20]. However, our patient's clinical characteristics are different from those previously studied since our patient has rheumatoid arthritis that requires chronic immunosuppression. In patients with progressive disseminated histoplasmosis who remain immunocompromised, it is suggested to treat indefinitely [1]. In a 2017 review of histoplasmosis in transplant recipients, it was recommended that patients with posttransplant histoplasmosis complete at least 12 months of therapy, show clinical resolution of signs and symptoms of infection, and have a urine and serum *Histoplasma* antigen <2 ng/mL before discontinuing therapy [21]. After discontinuing therapy, patients should be monitored for clinical signs of infection and have *Histoplasma* antigens measured every three months. In our patient, monitoring of *Histoplasma* antigens may be difficult to interpret as he had both a negative serum and urine *Histoplasma* antigen in the presence of active infection.

Although miliary infiltrates can be seen in disseminated histoplasmosis, our patient did not have other clinical manifestations to suggest he had disseminated disease. The patient was treated as a pulmonary histoplasmosis case with 1 week of amphotericin B with plans to complete at least one year of itraconazole. There are no definitive guidelines on when immunosuppressants should be restarted. For our patient, he was restarted on rheumatoid arthritis medications after completing 2 months of itraconazole.

## 4. Conclusion

This case highlights the challenges of diagnosing and managing miliary histoplasmosis in an immunocompromised patient. Pulmonary nodules in a miliary pattern can be seen in the setting of infections other than tuberculosis, and therefore other infectious possibilities should be considered in patients with miliary pulmonary nodules. Possible clinical presentations can resemble tuberculosis, and histoplasmosis should be considered in patients who appear to have tuberculosis, and mycobacteria cannot be isolated. There can be an absence of antigenemia and antigenuria in immunocompromised patients with histoplasmosis, and therefore histopathology and cultures remain the gold standards for making a definitive diagnosis. Our case also highlights the need for further studies to determine the optimal duration of histoplasmosis treatment in immunocompromised patients and to establish the shortest duration of antifungal therapy required for the safe reinstitution of immunosuppressants.

## Figures and Tables

**Table 1:** Comparison of cases of miliary histoplasmosis.

Year	Reference	Age (yrs)	Sex	Comorbidities	Symptoms	Chest imaging	Initial diagnosis	Final diagnosis	Method of diagnosis	Outcome
1983	Tong et al. [12]	45	F	None	Progressive weight loss, abdominal swelling, intermittent fever, nocturnal sweating	Chest X-ray: diffuse pulmonary miliary shadows with scattered nodular lesions	Miliary tuberculosis	Disseminated histoplasmosis with pulmonary involvement	Autopsy	Death
2012	Cormier et al. [13]	37	M	None	Fever, abdominal pain, diarrhea, weight loss, asthenia	Chest X-ray: diffuse bilateral opacities with a 1-2 mm radius simulating miliary tuberculosis	Miliary tuberculosis	Disseminated histoplasmosis with pulmonary involvement	BAL	Death
2013	Cottle et al. [14] *13 students with respiratory symptoms. 5 with confirmed histoplasmosis. 2 cases described here*	22	F	None	Fever, dry cough, chest pain, shortness of breath with exertion	Chest X-ray: diffuse miliary shadowing	Miliary tuberculosis	Pulmonary histoplasmosis	Serum antibodies	
		21	M	None	Productive cough, shortness of breath, night sweats	CT: mediastinal lymphadenopathy, bibasilar consolidation, bilateral pulmonary micronodules	Miliary tuberculosis	Pulmonary histoplasmosis	Serum antibodies	Survived
2015	Lakshman et al. [15]	25	F	HIV	Fevers, chills, cough, hematemesis, melena	CT: miliary nodules in both lungs	Disseminated tuberculosis	Disseminated histoplasmosis with pulmonary involvement	Bone marrow biopsy	Survived
2017	Our case	52	M	RA	Fevers, chills, cough, fatigue, night sweats, nausea, vomiting	CT: diffuse micronodularity	Miliary coccidioidomycosis	Miliary histoplasmosis	Left upper lobe tissue culture	Survived
